# The killer of Socrates: Coniine and Related Alkaloids in the Plant Kingdom

**DOI:** 10.3390/molecules22111962

**Published:** 2017-11-14

**Authors:** Hannu Hotti, Heiko Rischer

**Affiliations:** VTT Technical Research Centre of Finland Ltd., P.O. Box 1000, 02044 Espoo, Finland; hannu.hotti@gmail.com

**Keywords:** *Aloe*, alkaloids, coniine, poison hemlock (*Conium maculatum* L.), polyketides, *Sarracenia*, secondary metabolism, Socrates

## Abstract

Coniine, a polyketide-derived alkaloid, is poisonous to humans and animals. It is a nicotinic acetylcholine receptor antagonist, which leads to inhibition of the nervous system, eventually causing death by suffocation in mammals. Coniine’s most famous victim is Socrates who was sentenced to death by poison chalice containing poison hemlock in 399 BC. In chemistry, coniine holds two historical records: It is the first alkaloid the chemical structure of which was established (in 1881), and that was chemically synthesized (in 1886). In plants, coniine and twelve closely related alkaloids are known from poison hemlock (*Conium maculatum* L.), and several *Sarracenia* and *Aloe* species. Recent work confirmed its biosynthetic polyketide origin. Biosynthesis commences by carbon backbone formation from butyryl-CoA and two malonyl-CoA building blocks catalyzed by polyketide synthase. A transamination reaction incorporates nitrogen from l-alanine and non-enzymatic cyclization leads to γ-coniceine, the first hemlock alkaloid in the pathway. Ultimately, reduction of γ-coniceine to coniine is facilitated by NADPH-dependent γ-coniceine reductase. Although coniine is notorious for its toxicity, there is no consensus on its ecological roles, especially in the carnivorous pitcher plants where it occurs. Lately there has been renewed interest in coniine’s medical uses particularly for pain relief without an addictive side effect.

## 1. Introduction

Coniine is an alkaloid which is known to be present in a diversity of plants, including monocots (*Aloe* [1,2,3,4]) and dicots (*Conium* (e.g., [5]) and *Sarracenia* [6,7]). Coniine holds two records in chemistry in that it constitutes the first alkaloid the structure of which was fully resolved, and that has been chemically synthesized. Poison hemlock, and thus coniine, have historically been used as medicine but not anymore due to a narrow treatment window. Its mode of action in the nervous system is to block nicotinic acetylcholine receptors which, in the worst case, can lead to death by suffocation. Coniine’s ecological role in plants, on the other hand, is not yet fully revealed, especially in insectivorous *Sarracenia* plants.

Previously, the poison hemlock and coniine literature has been reviewed by López et al. [8], Vetter [9] and Reynolds [10]. Since then, there have been many advances related to the biosynthesis, occurrence in plants, molecular diversity and biological interactions, justifying an updated summary.

## 2. Hemlock Alkaloids and Their Chemistry

Piperidine alkaloids are characterized by a six-membered saturated heterocyclic ring, i.e., piperidine nucleus. Over 700 members of this alkaloid class are currently known [11].

**Figure 1:**
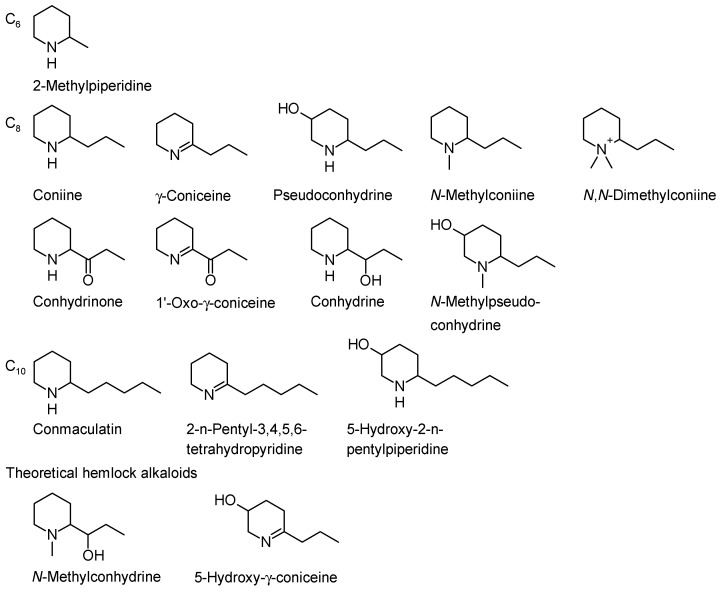
Classification of hemlock alkaloids naturally occurring in poison hemlock (*Conium maculatum* L.), *Sarracenia* spp. and *Aloe* spp. according to their carbon number. Theoretical hemlock alkaloids are *N*-methylconhydrine [12] and 5-hydroxy-γ-coniceine [13]. Reproduced from [14].

Currently, there are thirteen alkaloids known from poison hemlock (in the following text referred as hemlock alkaloids) (Figure 1), which can be classified according to the number of carbon atoms in their backbone. The simplest category, C_6_, comprises only one member, 6-methylpiperidine. The C_8_-category includes nine members. The C_10_-category is the most recent in hemlock alkaloids. Its first members were discovered in 1997. In the literature, there are hints that even more alkaloids could exist. The following list describes all known, and two theoretical hemlock alkaloids.

**2-Methylpiperidine**. C_6_H_13_N. MW 99.17 g/mol. CAS number 109-05-7. Also known as 2-pipecoline, α-pipecoline, α-methylpiperidine. The boiling point is 120 °C [15] and the melting point is −4 °C [16]. 2-Methylpiperidine is an alkaloid found from poison hemlock (*Conium maculatum* L.) [5,17].

**Coniine**. C_8_H_17_N. MW 127.23 g/mol. CAS number 458-88-8. Also known as 2-propylpiperidine, cicutine, conicine. The boiling point is 166 °C and the melting point −2 °C [10]. Coniine was first isolated from poison hemlock in 1826 by Giseke [18]. Its structure was established in 1881 by Hofmann [19] and soon thereafter, in 1886, it was synthesized by Ladenburg [20], thus making it the first alkaloid to be structurally characterized and chemically synthesized. Coniine has a stereocenter at position C-2, leading to two stereoisomers, (*S*) and (*R*), of which the former is the naturally occurring [10].

**γ-Coniceine**. C_8_H_15_N. MW 125.21 g/mol. CAS number 1604-01-9. Also known as 2-propyl-1,4,5,6-tetrahydropyridine. The boiling point is 171 °C [10] and the predicted melting point is −4.56 °C [21]. γ-Coniceine was isolated from poison hemlock by Wolffenstein [22]. He also determined the chemical formula, but the structure was determined as late as 1961 by Beyerman et al. [23] by hydrogen nuclear magnetic resonance and infrared. The alkaloid was first synthesized by Gabriel [24]. γ-Coniceine is the principal alkaloid in leaves and young tissues of poison hemlock [5].

**Pseudoconhydrine**. C_8_H_17_NO. MW 143.23 g/mol. CAS number 140-55-6. Also known as 6-propyl-3-piperidinol, Ψ-conhydrine. The predicted boiling point is 246 °C [21] and the melting point is 105 °C [10]. Pseudoconhydrine was isolated from poison hemlock by Merck [25]. Its structure was finally determined by Yanai and Lipscomb [26]. It is an isomer of conhydrine with a hydroxyl group at C-5. This alkaloid is usually present in poison hemlock as a minor alkaloid but in one American strain it is reported to be a major component and it originates from γ-coniceine [13].

***N*-Methylconiine**. C_9_H_19_N. MW 141.25 g/mol. CAS number 35305-13-6. Also known as 1-methyl-2-propylpiperidine, methylconiine. The predicted melting and boiling points are −6.2 °C and 177.6 °C, respectively [21]. It was first synthesized from coniine and its formula was determined by von Planta and Kekulé [27]. The alkaloid’s structure was determined by Wolffenstein [28] from poison hemlock extract.

***N*,*N*-Dimethylconiine**. C_10_H_22_N. MW 156.24 g/mol. CAS number 329270-32-8. Also known as 1,1-dimethyl-2-propylpiperidinium. The predicted melting and boiling points are 144.4 °C and 383.2 °C, respectively [21]. It was isolated from *Aloe sabaea* [3].

**Conhydrinone**. C_8_H_15_NO. MW 141.21 g/mol. CAS number 97073-23-9. Also known as 1-(2-piperidynyl)-1-propanone. The predicted melting point is 34.9 °C [21] and the boiling point is 94 °C [29]. It was isolated from poison hemlock by Leete and Olson [30]. They also showed that biosynthetically conhydrinone originates from γ-coniceine.

**1′-Oxo-γ-coniceine**. C_8_H_13_NO. MW 139.19 g/mol. CAS number 80933-75-1. Also known as 1-(3,4,5,6-Tetrahydro-2-pyridinyl)-1-propanone. The predicted melting and boiling points are 24.9 °C and 213 °C, respectively [21]. It was found from poison hemlock by Holstege et al. [17].

**Conhydrine**.C_8_H_17_NO. MW 143.23 g/mol. CAS number 495-20-5. Also known as 2-(1-hydroxypropyl)piperidine, α-ethyl-2-piperidinemethanol, 2-(α-hydroxypropyl)-piperidine. The melting point is 121 °C [10] and the predicted boiling point is 241 °C [21]. It was found from poison hemlock in (+) form by Wertheim [31].

***N*-Methylpseudoconhydrine**. C_9_H_19_NO. MW 157.25 g/mol. CAS number 78962-69-3. Also known as 1-methyl-6-propyl-3-piperidinol. The melting point is 157 °C [32] and the predicted boiling point is 238.6 °C [21]. The alkaloid was found in a South-African yellow-flowered *Conium* sp. [32].

**Conmaculatin**. C_10_H_21_N. MW 155.28 g/mol. CAS number 33354-97-1. Also known as 2-pentylpiperidine. The predicted melting point is 30 °C [21] and the boiling point is 207–208 °C [33]. The alkaloid was first found from Serbian poison hemlock [34].

**2-n-Pentyl-3,4,5,6-tetrahydropyridine**. C_10_H_19_N. MW 153.26 g/mol. CAS 5832-23-5. The predicted melting and boiling points are 8.4 °C and 212 °C, respectively [21]. The alkaloid was discovered from poison hemlock [35]. The compound has also been found from fire ant (*Solenopsis*) species [36].

**5-Hydroxy-2-n-pentylpiperidine**. C_10_H_21_NO. MW 171.28 g/mol. CAS number 220088-35-7. Also known as 6-pentyl-3-piperidinol. The predicted melting and boiling points are 66 °C and 280 °C, respectively [21]. It was found in poison hemlock [36].

**Other possible hemlock alkaloids**. Research performed by Cromwell [5] and Fairbairn and Suwal [37] indicated that poison hemlock contains alkaloids which have larger molecular size and are more polar. Castells et al. [38] found an unknown alkaloid from poison hemlock. Its MS fragmentation pattern (*m*/*z*: 125 (M^+^, 1), 124 (6), 110 (18), 97 (100), 96 (31), 82 (9), 69 (7), 55 (15)) is similar to that of γ-coniceine, thus making it a possible γ-coniceine isomer, but the location of the double bond is unknown. Roberts [12] postulated the possible existence of *N*-methylconhydrine (Figure 1) in poison hemlock after a series of enzymatic studies. Leete and Adityachaudhury [12] theorized that 5-hydroxy-γ-coniceine could be an intermediate product in the biosynthesis route of pseudoconhydrine from γ-coniceine.

Chemical synthesis of piperidines, e.g., coniine, has been achieved and is constantly further developed based on various reaction types such as Mannich-type reaction, Michael addition, ring-closing metathesis, iminium ion cyclization, Diels–Alder reaction, Pd^II^-catalyzed reaction of nitrogen nucleophile [39], intramolecular N–C bond formation, C–C bond formation, intermolecular reactions and modification of six-membered nitrogen heterocycles [40].

## 3. Plant Containing Hemlock Alkaloids

Piperidine alkaloids are found in many genera, for example, *Nicotiana*, *Conium*, *Lobelia*, *Pinus*, *Punica*, *Duboisia*, *Sedum*, *Withania*, *Carica*, *Hydrangea*, *Dichroa*, *Cassia*, *Prosopis*, *Genista*, *Ammodendron*, *Lupinus*, *Liparia* and *Collidium* [41]. Fire ants (*Solenopsis* sp.), as an example outside the plant kingdom, also contain simple piperidine alkaloids [36]. The occurrence of hemlock alkaloids among plants is restricted to few but unrelated genera.

### 3.1. Conium *sp.*

**Figure 2:**
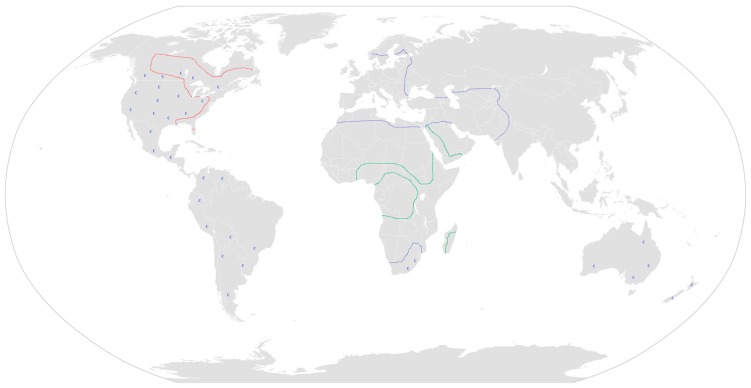
Distribution map of plants containing hemlock alkaloids. *Aloe* in green, *Conium* in blue and *Sarracenia* in red. **C** for poison hemlock as an introduced weed.

The genus *Conium* has five members, of which four (*C. chaerophylloides*, *C. fontanum*, *C. hilliburttorum*, *C. sphaerocarpum*) are South African [42]. They belong to the family Apiaceae, which comprises 434 genera and 3780 species depending on classification [43]. The most investigated member of the genus is poison hemlock (*C. maculatum*). Poison hemlock is native to Europe, northern Africa, and western Asia. It has also been introduced to North America, South America, Australia and New Zealand (Figure 2) [44]. The old Roman name for poison hemlock was *cicuta*, which is used nowadays for water hemlock (*Cicuta virosa* L.). The modern name is derived from the Greek word ‘Konas’, ‘to whirl about’ since consumption causes ataxia, tremor, and convulsions. The Latin word *maculatum* means spotted, and indeed the plant has reddish spots on the stem and the leaf stalk. Hemlock has been written hemlic/hymelic (Anglo–Saxon), hymlice, hymlic, hemeluc, hemlake, hemlocke and hemlock (first used in William Shakespeare’s play ‘Life of Henry the Fifth’) [9]. Common names for the plant (Table 1) include poison parsley, carrot fern, conium, spotted hemlock, spotted cowbane, spotted corobane, carrot weed, California or Nebraska fern, musquash root and poison root [45,46,47,48].

**Figure 3:**
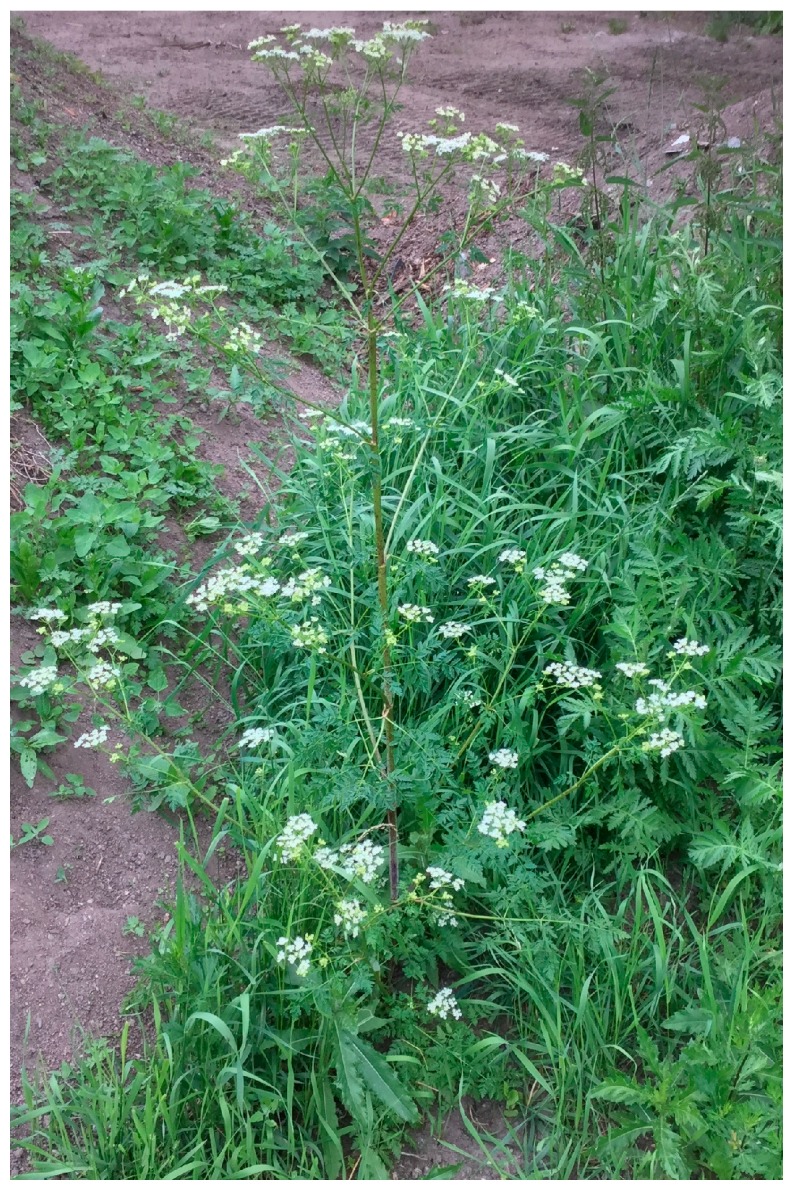
Poison hemlock (*Conium maculatum* L.).

Poison hemlock (Figure 3) (2n = 22) is a herbaceous biennial plant, but it can also be a winter annual or a short-lived perennial. The stem is stout and can achieve a height of 1–2 m. It is straight, branched, mainly in the upper part. Its texture is smooth and colored pale green with purple spots, which are the most important key identification of the species. The basal part is 4–8 mm thick and terete or angled. The cross-section of the stem is hollow or rarely solid, except at the nodes where it has fine, shallow, longitudinal ridges. The inflorescences are large, compound umbels which are open and 4–6 cm across. The terminal umbel, which is situated at the top, blooms first. The flowers are small in large, loose clusters with a circle of narrow bracts at the base. The poison hemlock has five petals, which are white, incurved and devoid of sepals. The bracts are involucre lanceolate, acuminate and inconspicuous. The fruit is a schizocarp, oval to circular in outline and forms a compound of two grey or brown mericarps (seeds). The seeds are narrowly ovate, 1.2–2 mm wide, 2–3 mm long, with a slightly extended apex, the dorsal side strongly convex with 5 prominent wave ridges from top to bottom and they weight about 0.5 mg. Its taproot is long, 5–11 mm thick, whitish and usually unbranched. The leaves are present as a rosette from the crown and alternately on the stem. They are 20–40 cm long with 3 or 4 times pinnate leaf compounds and their segments are toothed or deeply cut. The base of the petiole tends to sheath the stem and is shorter on upper leaves [44,49].

All tissues of poison hemlock contain alkaloids in abundance [50]. The main alkaloid of flower buds and flowers is γ-coniceine, which is transformed during the fruit development into coniine and further into *N*-methylconiine, which are the main alkaloids in mature fruit [5]. The volatile alkaloids in flowers may attract pollinators [51].

There is a “coniine layer” in fruits which starts to develop during week 3 after fertilization. By week 7 the walls are completely thickened. During week 3 the fruits also contain the maximum amount of alkaloids, which can be as much as 3% of dry weight [5,52]. The role of alkaloids in fruits may be related to protection of seeds before germination, as the coniine layer is lost during germination [51]. There are secretory structures, called ‘vittae’, in seedlings, vegetative organs, flowers and fruits, which are possible sites for alkaloid biosynthesis [50].

Poison hemlock is a cross-breeding species [53]. The plant is a prolific seed producer with 1700–39,000 seeds per plant and can dominate small areas if left unchecked, at the same time creating a seed bank [54,55]. Seed dispersal occurs from mid-September to late February, as the stalks persist through winter, and by late December 95% of the seeds have been dispersed [55,56]. Seeds do not have a dormancy restriction, which allows them to germinate as soon as the prevailing conditions permit. Depending on the year, 40–85% of seeds will germinate when there is a suitable temperature difference. Generally, poison hemlock seeds remain viable for 3–6 years [48,55].

Poison hemlock belongs to a group of widely spread weeds [9]. It is a pioneer plant and thus an invasive species [48]. The plant’s invasiveness is due to its ability to grow in very different locations, such as in cultivated fields, waste areas, along ditch banks or fences, around barnyards, waterways and along roadsides [54,57]. The general habitat is moderately dry, mull-rich and nutrient-rich bare soil with good moisture in the light-open area [49,54]. The plant tolerates a high level of heavy metals (arsenic, cadmium, lead) in soil [53].

Poison hemlock can contaminate harvested hay, as it grows in alfalfa fields. It also grows in grass pastures and meadows, causing a risk of poisoning to grazing livestock, especially in the spring when there is little else to feed on and poison hemlock is one of the first plants to emerge (in the USA) [54]. Thus, poison hemlock is part of the overall problem caused by introduced weeds, which result in about $1–2 billion/year losses in forage crops in the USA [58,59].

Parts of poison hemlock have been confused with many edible Apiaceae plants; its leaves with parsley (*Petroselinum crispum* (Mill.) Fuss), roots with parsnip (*Pastinaca sativa* L.) and seeds with anise (*Pimpinella anisum* L.) [9,57,60]. Other mix-ups are with e.g., *Chaerophyllum* sp., *Anthriscus sylvestris* (L.) Hoffm and *Aethusa cynapium* L. [49]. Key characteristics to distinguish poison hemlock from other Apiaceae, e.g., wild carrot, *Daucus carota* L., are the unbranched stems with red spots and the absence of hairs on stems and leaves. When crushed, the pungent smell is very intense [49].

In order to prevent spreading of invasive poison hemlock, the plants should be eradicated as soon as they are noticed. A 3–6-year approach is needed, as the plant stand cannot be effectively controlled in one year [48]. For chemical control of poison hemlock, suitable compounds include hexazinone (active over 90%); metribuzin (over 90%); terbacil (over 90%); glyphosate at 1.1 kg/ha (97–98%); and 2,4-dichlorophenoxyacetic acid (2,4-D) at 1.1 kg/ha (97–98%) [48,57,61,62]. The spraying should be performed when seedlings emerge or before the flowering stalk begins to develop in order to stop the invasion [57]. Physical control should be applied before the plants produce seeds. Manual methods include hoeing, digging, cutting and grubbing. Recommended mechanical methods are mowing and tillage. For biological control, the hemlock moth (*Agonopterix alstroemeriana* Clerk) may be used [48].

### 3.2. Aloe *sp.*

The genus *Aloe* is a member of the family Xanthorrhoeaceae [63]. About 400 species are included in the genus, which is native to most of Africa south of the Sahara Desert, the Arabian Peninsula, Madagascar and several smaller islands in the Indian Ocean [64]. The habitat of *Aloe* is generally an arid or semi-arid region, more specifically dry forests, open woodland, grassland, bare rock surfaces and cliff faces [65]. Humans have used aloes for several millennia at least, as *A. vera* (L.) Burm.f. was used as a medicine in China and India about 2400 years ago [66]. Nowadays they are mainly ornamental plants in warm climates. In traditional medicine, the aloes have a role in treating burns, skin disorders and as a purgative. In cosmetics, extract of aloe is used e.g., in soaps. In many African cultures cut aloes are dried and used as such in dyes. Poisonous *A. ruspoliana* Baker is used to kill hyenas in eastern Africa.

*Aloe* species are rich in secondary compounds. These include various chromones, anthraquinones, anthrones, coumarins, pyrones, flavonoids and sterols [67]. Very few alkaloids have been described from the genus, namely the purines hypoxanthine and xanthine from *A. ferox* Mill. [68], and the tyramine-derivated phenylethylamines in 18 *Aloe* species [2]. Piperidine alkaloids are present in twelve species (Table 2) [1,2,3]. *A. globuligemma*, used as a herbal medicine, has caused deaths in rural Africa [69]. Parry and Matambo [70] studied the toxicity of *A. globuligemma* as it is used as a traditional herbal medicine in Zimbabwe. Its crude extract has LD_50_ < 250 mg/kg on mice and the toxic symptoms were reported to be similar to those of poison hemlock poisoning.

### 3.3. Sarracenia *sp.*

Sarraceniaceae is a New World carnivorous plant family. It includes three genera, *Darlingtonia*, *Heliamphora,* and *Sarracenia.* In *Sarracenia*, there are 8–11 species depending on the classification [71,72]. The native range of *Sarracenia* is the eastern seaboard of North America. The native habitats are nutrient-poor, acidic and wet environments comprising swamps, fens and grassy plains. All *Sarracenia* species are insectivorous i.e., they attract, capture and digest insects to supplement their nutrient uptake. A common feature of all *Sarracenia* is that they lure insects to their elongated tubular leaves. *S. psittacina* Michx. hides the entry/exit hole using multiple translucent false entries so that trapped insects finally tire and die. Other *Sarracenia* species utilize downward pointing hairs and waxy substances in their pitchers to trap insects.

Initially, Mody et al. [6] reported 5 mg of coniine from 45 kg fresh plant material via steam distillation. Hotti et al. [7] confirmed from the occurrence in S. flava and additionally detected it in *S. alata*, *S. leucophylla*, *S. minor*, *S. oreophila*, *S. psittacina*, *S. purpurea* and *S. rubra*. In all cases coniine is present in low amounts in the studied plant material. Mody et al. [6] tested the isolated coniine with fire ants and found that the compound paralyzed them. Harborne [73] suggested that coniine not only paralyzes insects but also entices them into the pitcher. In contrast to *Conium* [50], it is still unknown where coniine is biosynthesized in *Sarracenia* sp. i.e., whether it is in the lid, hairs, extrafloral nectar, mouth, middle or lower part of the pitcher.

### 3.4. Other Plants Possibly Containing Hemlock Alkaloids

There are speculations that other plants contain hemlock alkaloids, too, but detailed investigations are missing. Such plants include upright spurge (*Euphorbia stricta* L.) and crown imperial (*Fritillaria imperialis* L.), both of which have reportedly a characteristic mousy smell when crushed [10]. *Fritillaria* spp. definitely contains alkaloids, but so far only typical steroid-based alkaloids were found [74]. Hébert and Haim [75] speculated that different aroids (*Amorphophallus* sp., *Arisarum* sp., *Arum* sp., *Caladium* sp.) could contain hemlock alkaloids. Raffauf [76] continued this list with the genera *Sarcolobus, Punica,* and *Parietaria*. Power and Tutin [77] examined fool’s parsley (*Aethusa cynapium*) and reported coniine or a related alkaloid. Later research has shown that the then identified compound is aethusin (cynapine), a polyacetate [78].

**Figure 4:**
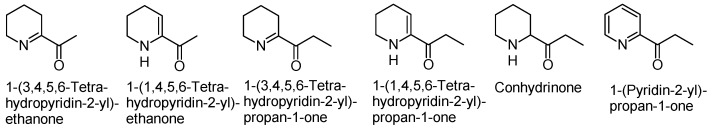
Alkaloids of *Semnostachya menglaensis* Tsui [79].

Interestingly, *Semnostachya menglaensis* Tsui (Apocynaceae), a rare plant from the Yunnan province of China, reportedly contains 1′-oxo-γ-coniceine as a major component and conhydrinone in its volatile oil. The plant contains four other simple alkaloids (Figure 4) [79]. Conhydrine has been reported from Bermuda grass (*Cynodon dactylon* (L.) Pers.), Poaceae [80]. Another report indicates the presence of the same alkaloid in lemon balm (*Melissa officinalis* L.), Lamiaceae [81]. Coniine, *N*-methylconiine, 1-methyl-2-butylpiperidine and 1-methyl-2-pentylpiperidine have been reported from *Pimpinella acuminate* (Edgeworth) C. B. Clarke [82].

## 4. Biosynthesis of Hemlock Alkaloids

Robinson [83] suggested that piperidine alkaloid biosynthesis starts from the amino acid lysine. Typically, the amino acid is first processed into cadaverine and then Δ^1^-piperideine from which several piperidine alkaloids are derived [84,85]. This single idea has later been expanded by recognizing that besides lysine, the carbon backbone of piperidine alkaloids can also originate from acetate, mevalonate or monoterpenes (e.g., alkaloid skythanthine) [86].

Poison hemlock is the only plant species in which hemlock alkaloid biosynthesis has been studied. Leete [87] fed uniformly labeled [^14^C]-l-lysine, [2-^14^C]-dl-lysine and [1,5-^14^C]-cadaverine to poison hemlock. These feedings resulted in negligible activity in the alkaloids. However, Cromwell and Roberts [88] fed uniformly labelled [^14^C]-l-lysine, [^14^C]-Δ^1^-piperideine and [^14^C]-Δ^1^-piperideine-2-carboxylic acid together with [6-^14^C]-dl-α-aminoadipic acid, [1,5-^14^C]-cadaverine and [2-^14^C]-propionate, which were incorporated into γ-coniceine. Leete [87,89] interpreted his results obtained with uniformly labeled [^14^C]-lysine so that lysine underwent metabolism in the plant, finally ending up as acetate, which was ultimately incorporated into γ-coniceine and then into coniine. However, because lysine was not incorporated directly into coniine, Leete [87,89] proposed an alternative hypothesis that acetate, rather than lysine, could function as the carbon source for coniine.

**Figure 5:**
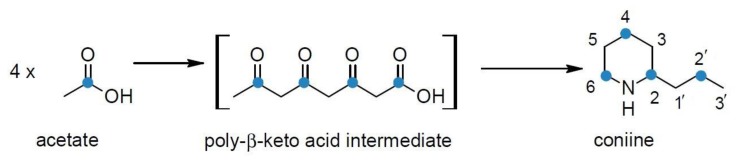
Poison hemlock plants were fed sodium [1-^14^C]-acetate and the radioactive carbon (blue dots) was found in the even-numbered carbons of coniine [87,89].

In order to verify this, Leete [87,89] fed poison hemlock plants sodium [1-^14^C]-acetate and the activity from labeled acetate was detected in the carbons of coniine and conhydrine (Figure 5). Via systematic degradation, it was indeed established that almost all the activity remained in the even-numbered carbons and was equally distributed between these positions (C-2 26%, C-3 1.6%, C-4 22%, C-5 1%, C-6 24%, C-1′ 1.3%, C-2′ 22%, C-3′ 1.6%). He postulated that the possible formation route could be derived from one acetyl-CoA and three malonyl-CoAs. Thus, the carbon backbone of coniine would originate from acetates via the intermediary of a poly-β-keto acid (polyketide) [10,51,87,89].

In order to get further proof, Leete [90] fed poison hemlock plants labeled sodium [1-^14^C]- and [2-^14^C]-octanoate. The plants were harvested after seven days and the labeled carbon was found in γ-coniceine. He postulated that octanoic acid must have been activated to 5-keto-octanoic acid and/or cleaved to acetyl-CoA via the Krebs cycle and processed further into γ-coniceine. In other words, octanoic acid is not used directly as such but via oxidation. In the next stage Leete and Olson [91] fed plants sodium [1-^14^C]-acetate, [1-^14^C]-octanoic acid, [6-^14^C]-5-keto-octanoic acid and [6-^14^C]-5-keto-octanal, and the plants were harvested after 24 h. The incorporation rates were 0.009% for [1-^14^C]-acetate, 0.07% for [1-^14^C]-octanoic acid, 0.61% for [6-^14^C]-5-keto-octanoic acid and 1.1% for [6-^14^C]-5-keto-octanal. They noticed that octanoic acid is not the preferred precursor for the alkaloids as it shows a poor incorporation rate. The compound could, however, lead to the formation of 5-keto-octanoic acid, which probably happens before alkaloid formation, as its activity was in the C-1′ carbon of formed alkaloids. Therefore, they claimed that 5-keto-octanal and 5-keto-octanoic acid are the most probable precursors in the alkaloid biosynthesis [30]. They suggested that 5-keto-octanal goes through transamination to form γ-coniceine. The key to hemlock alkaloid production could therefore be the availability of 5-keto-octanal [51]. Even though [1-^14^C]-hexanoic acid was incorporated in coniine at a poor rate this could be explained by chain elongation in the form of hexanoyl-CoA with malonyl-CoA [84]. Accordingly, 2-methylpiperidine biosynthesis could proceed in an analogous manner from 5-ketohexanoic acid.

The problem of identifying the enzyme responsible for carbon backbone formation of coniine alkaloids was recently approached by isolating and characterizing full-length genes of type III polyketide synthases (PKS) expressed in tissues of poison hemlock. Hotti et al. [92] isolated CPKS5 which is expressed in stem, flower and developing fruit, and fed different aliphatic starter-CoAs in in vitro enzymatic tests to follow up on Leete’s hypothesis. The tests indicated that the order of preference for starter units was as follows: butyryl-CoA (at pH 6.2 with k_cat_/K_m_ 1595 s^−1^ M^−1^), hexanoyl-CoA, acetyl-CoA and finally octanoyl-CoA. CPKS5 exhibits seven amino acids changes in the active site when compared to a typical chalcone synthase [93]. Based on these observations it was suggested that coniine’s carbon backbone is formed from one butyryl-CoA and two malonyl-CoAs.

Leete’s hypothesis [87,89], proposing a tetraketide backbone of coniine formed from one acetyl-CoA and three malonyl-CoAs, remains unsupported because a PKS favoring acetyl-CoA as a starter could not be identified. On the other hand, the substrate preference of CPKS5 correlates with the alkaloid presence in poison hemlock. It is tempting to speculate that the same enzyme could be responsible for the polyketide formation of all C_6_, C_8_ and C_10_ alkaloids in the plant. Such a scenario would involve condensation of either acetyl-, butyryl-, or hexanoyl-CoA with two malonyl-CoAs to form a triketide. C_8_-alkaloids are the most common (e.g., [5]), consistently with butyryl-CoA being the favored starter among those tested. C_10_-alkaloids are minor alkaloids. Conmaculatin is a relatively new finding [36] that probably escaped earlier analyses due to its low amounts. Hexanoyl-CoA is a suitable starter but may not be preferred naturally. The only C_6_-alkaloid, 2-methylpiperidine, has occasionally been reported [5,17], however, CPKS5 does not utilize acetyl-CoA very well under the tested conditions [92]. According to the in vitro substrate tests there could theoretically be C_12_-alkaloids in poison hemlock, but hitherto these have not been observed.

**Figure 6:**
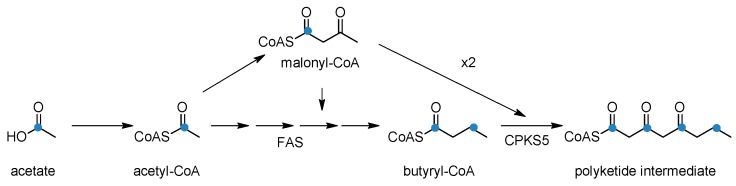
An alternative explanation of how the labeling pattern of ^14^C described by Leete [8,87] could be explained. Blue dots present the labeled carbon and FAS is fatty acid synthase.

In the light of the current knowledge, Leete’s labeling results in planta [87,89] could be interpreted so that fed acetate is first activated into acetyl-CoA, part of which is further processed into malonyl-CoA. Acetyl-CoA is then elongated with malonyl-CoA by fatty acid synthase to form butyryl-CoA [94], resulting in the labeling of even-numbered carbons when using ^14^C-labelled acetate substrate. Then butyryl-CoA is elongated twice with malonyl-CoA containing ^14^C originating from the fed acetate (Figure 6).

**Figure 7:**
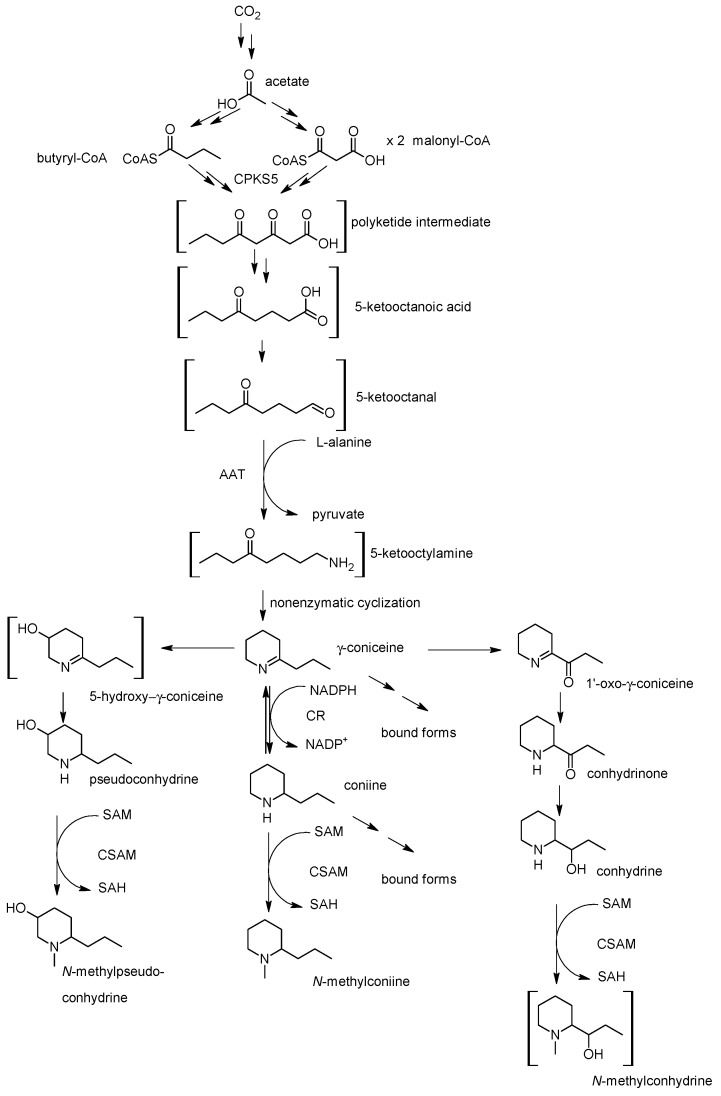
Biosynthetic pathway scheme of coniine in poison hemlock based on [12,14,30,52,87,89,91,92,95,96,100,101,102,103,104,105]. Hypothetical intermediates and alkaloids are shown in brackets. Abbreviations: AAT l-alanine:5-keto-octanal aminotransferase, CSAM *S*-Adenosyl-L-methionine:coniine methyltransferase, CR γ-coniceine reductase, CPKS5 *Conium* polyketide synthase 5.

Further downstream the series of steps are characterized on the enzymatic level. Nitrogen of hemlock alkaloids is introduced in a transaminase-catalyzed reaction between 5-keto-octanal and l-alanine by l-alanine:5-keto-octanal aminotransferase (AAT) (Figure 7) [95]. The enzyme has no reverse activity, i.e., the reaction is unidirectional; l-alanine:5-ketooctanal to pyruvate:γ-coniceine with an activity of 10 U/mg protein. Serine, glutamic acid, 3-aminobutyric acid and 6-amino hexanoic acid can also function as nitrogen donors for AAT [96]. The transaminase has two isozymes, A and B, with the same molecular size, 56.23 kDa [97]. Isozyme A is mitochondrial [98] and its K_m_ for 5-keto-octanal is 1.6 mM and for l-alanine 27 mM, with a pH optimum of 7.5–8.5 [97]. Isozyme B is chloroplastic [98], and its K_m_ for 5-keto-octanal is 0.14 mM and for l-alanine 55 mM, with a pH optimum of 8.5 [97]. Roberts [98] suggested that especially the isozyme B would be the transaminase responsible for alkaloid formation. An AAT isolated from spinach (*Spinacia oleracea* L.) leaves is also capable of forming γ-coniceine from 5-keto-octanal with l-alanine [99]. The cyclization after transamination is a non-enzymatic reaction [96,99].

γ-Coniceine is a precursor of coniine [13,52,100,101,103], which is formed by γ-coniceine reductase (CR). The enzyme was isolated from metabolically active leaves and unripe fruits of hemlock, in which coniine is a major alkaloid. The CR is NADPH-dependent [105] and catalyzes the formation of the (*S*)-isomer of coniine [30]. Conhydrinone [30] and pseudoconhydrine [13] are also derived from γ-coniceine.

*N*-Methylation of coniine leads to *N*-methylconiine [100,101]. The hemlock plants were fed [methyl-^14^C]-l-methionine and the methyl group was incorporated into *N*-methylconiine [106]. The enzyme behind the reaction is *S*-adenosyl-l-methionine:coniine methyltransferase (CSAM) [104], which was isolated from unripe fruits. The actual donor of the methyl group is *S*-adenosyl-l-methionine. The enzyme’s optimal reaction rate is 140 nmol coniine/h/mg protein with K_m_ 1.55 mM and its optimal pH is 8.2. The enzyme accepts coniine, conhydrine and pseudoconhydrine as substrates to produce *N*-methylated alkaloids. CSAM works best with pseudoconhydrine, followed by coniine and most poorly with conhydrine [12].

As mentioned before (Section 3.1) seeds are major accumulation sites for hemlock alkaloids. Otherwise, Cromwell [5] deduced that the biosynthesis of alkaloids is more likely to occur in shoots than in roots. This conclusion was based on the fact that the root sap of a decapitated plant was devoid of alkaloids, and there was no accumulation over a period of one week. Fairbairn and Suwal [37] noted that roots of young seedlings did not contain alkaloids, in contrast to the roots of second-year plants before spring growth but during the growth season; alkaloids were not detected in their roots either. [^14^C]-Labelled alkaloids in seeds were not found in germinating cotyledons, as γ-coniceine of seedlings is synthesized de novo [12]. The key enzymes of alkaloid production (AAT, CR and CSAM) are active during leaf expansion. This activity ceases when the leaf has matured and a similar situation prevails in the fruits [51]. Interestingly, green callus of poison hemlock does not contain any alkaloids even when elicitated [107,108].

In poison hemlock, the switch from γ-coniceine to saturated alkaloid accumulation may be associated with active growth in a reversible fashion [52,103]. Coniine content can vary by over 100% and γ-coniceine by over 400% during the daytime. The amount of γ-coniceine peaks around midday, when coniine is absent. There are also hourly and daily changes in alkaloid concentrations [37]. Roberts [98] suggested that there is a close link between illumination and alkaloid production. Biosynthesis of pseudoconhydrine is apparently dependent on environmental factors, as the same variety produced conhydrine outdoors and pseudoconhydrine in a greenhouse [13]. γ-Coniceine is the dominant alkaloid during the rainy season and coniine in the dry season [52]. Lang and Smith [35] concluded that alkaloid production varies according to temperature and moisture conditions.

Another reason for the alkaloid fluctuation could be that hemlock alkaloids are coupled to an oxidation-reduction mechanism [52]. γ-Coniceine and coniine could have a similar function as NAD^+^ and NADH in poison hemlock [10,103]. However, increased alkaloid production is associated with general improvement in growth and vigor.

In the poison hemlock plant, especially the fruit, the alkaloids accumulate over time and yield γ-coniceine, coniine and some other alkaloids [102,103]. Coniine alkaloids are quite stable once they are formed, at least for nine days [30], and are present in non-volatile form [109].

## 5. Biological Activity of Hemlock Alkaloids

### 5.1. Mode of Action of Hemlock Alkaloids in Organisms

Hemlock alkaloids constitute neurotoxins and teratogens [10,110]. They affect the mammalian respiration so that it is first stimulated, then depressed, becoming cyanosed, and causing respiration failure [111]. Coniine is a nicotinic acetylcholine receptor (nAChR) antagonist. Its teratogenic action may be related to its ability to activate (stimulate) and subsequently, desensitize (depress) nAChRs, as this leads to inhibition of fetal movement [110].

One of the first studies on the biological effects of coniine in the body was performed in 1898 by Moore and Row [112]. They injected 10–20 mg coniine subcutaneously into a frog weighing about 25 g. This led to complete muscular paralysis of the animal. When the nerves of the frog were stimulated with electricity, there was no response due to paralysis of the intramuscular part of the muscular nerves. The coniine poisoning first affected the peripheral nervous system. In a rabbit, 50–60 mg coniine applied to the superior cervical ganglion induced partial paralysis, which rapidly passed off. The authors also observed that coniine slowed down amphibian and mammalian hearts. Coniine caused threefold dilation of arterioles as compared to the normal diameter. In mammals, coniine caused a slight quickening and marked deepening of the respiration. Later, the breathing became slower and the individual breaths became shallower. When coniine (20–70 mg) was injected into a cat or a dog, their respiration became feebler and finally halted due to peripheral paralysis of the respiratory muscles. There was no clear result to indicate the fatal dose. Moore and Row [112] concluded that coniine has similar physiological roles to those of nicotine and piperidine, only with varying intensity.

Bowman and Sangvi [111] studied the effects of coniine, *N*-methylconiine, and γ-coniceine on the body. Coniine (15–50 μg/mL) and γ-coniceine (5–15 μg/mL) caused contraction of isolated guinea pig ileum and rabbit duodenum. The action arose from stimulation of parasympathetic ganglia. *N*-Methylconiine did not cause any effect on the tested material. Blood pressure dropped with a coniine dosage of 0.5–2 mg/kg, *N*-methylconiine 1–4 mg/kg and γ-coniceine 0.2–0.5 mg/kg. In isolated rabbit heart, a decrease in the strength of beating was recorded with coniine at a dosage of 2 mg, *N*-methylconiine at 4 mg, and γ-coniceine at 0.2 mg. Generally, hemlock alkaloids intravenously or intra-arterially caused a small increase in venous outflow in skeletal muscle blood flow. The authors observed that respiration was first stimulated and then depressed during their animal testing. Coniine caused reportorial stimulation and depression at dosages of 1–4 mg/kg, whereas for γ-coniceine a lower dosage of only 0.3–1 mg/kg was sufficient. *N*-Methylconiine had no stimulatory effect; only depression was recorded in high doses due to neuromuscular block. Small dosages of coniine (20–30 μg) or γ-coniceine (10–20 μg) slowly increased the respiratory rate and depth, larger dosages caused depression of respiration and later respiratory failure.

Coniine blocks transmission through the superior ganglion and the neuromuscular junction [113]. It blocks acetylcholine in nictitating membrane. In frog neuromuscular endplate, coniine first reduces the amplitude and then causes depolarization of the membrane. In cat’s spine, the alkaloid causes depression and excitement. The depression is manifested by a decrease of monosynaptic response and the depression of post-tetanic potentiation. The excitement is the production of spontaneous waves and discharges recorded from the ventral root and blockade of direct and recurrent postsynaptic inhibition.

Coniine’s half maximal inhibitory concentration (IC_50_) is 314 μM in rat diaphragm, 70 μM in chick leg muscle, 1100 μM in maternal rat brain, 820 μM in fetal rat brain and 270 μM in chick brain [114]. Its binding to neuroreceptors and acetylcholine-related enzymes is in the adrenergic receptor Alpha_2_ 260 μM, in the serotonin receptor 5-HT2 492.7 μM, and in the acetylcholine receptors muscarinic AChR 2071 μM and nAChR 19 μM, and butylcholine esterase 327.5 μM [115].

Coniine, γ-coniceine and *N*-methylconiine block or reduce the response of muscle to acetylcholine [109] due to alkaloid binding to the nicotinic receptor of neuromuscular cells [114,116]. Coniine also exerts inhibitory effects on nicotinic receptor-mediated nitrergic and noradrenergic transmitter response in rat anococcygeus muscle via the inhibition of presynaptic nAChR [117]. When compared to different alkaloids in nAChR of fetal rat muscle, the efficiency is, from the highest to the lowest: nicotine > coniine > tubocurarine > lobeline [118]. The toxicities of coniine enantiomers on human tumour cell line TE-671 expressing human foetal nAChR are, in order from the highest to the lowest: γ-coniceine > (−)-coniine > (−)-*N*-methylconiine > (±)-coniine > (±)-*N*-methylconiine > (+)-coniine > (+)-*N*-methylconiine [116,119]. The exact values for different coniine enantiomers are presented in Table 3. (−)-Coniine elicits more effectively electrical changes in TE-671 cells. It also inhibits fetal movement in goats [120].

The hemlock alkaloids’ effects partly resemble those of curare [110,121] and nicotine in both the central and peripheral nervous systems [111]. Coniine potentiates morphine’s analgesic activity [122]. Hemlock alkaloids have analgesic (pain relieving) and anti-inflammatory activity in rats at 200 mg/kg (total alkaloids) [123]. Coniine has antinociceptive activity (preventing transmission of harmful signals in the nervous system) via nicotinic receptors at a dosage of 20 mg/kg in mice [122]. Conmaculatin has a strong peripheral and central antinociceptive activity in mouse over a dosage range of 10–20 mg/kg [34].

Little is known about how hemlock alkaloids are metabolized in the mammalian system(s) [86]. The microsomes of rat and chick liver did not biotransform coniine during testing (15 min). In insects, cytochrome P450 is involved when piperidine alkaloids are detoxified [124]. Another route to remove coniine is excretion (via urine or feces) rather than biotransformation (metabolism or catabolism) [114]. In rats, piperidine alkaloid piperine is excreted via feces (3%) and the rest of it via catabolism in the liver in the form of conjugated uronic acids, sulfates and phenols which are excreted via urine [125].

### 5.2. Pharmacology of Poison Hemlock and Hemlock Alkaloids

Poison hemlock has been used as a medicine externally to treat herpes, erysipelas (also known as *Ignis sacer*, holy fire, and St. Anthony’s fire; a bacterial skin infection caused by *Streptococcus pyogenes* Rosenbach) and breast tumors. The Greek and Arabian physicians used the plant to cure indolent tumors, swellings and pains in the joints. Poison hemlock was used in antiquity to wither testicles and to shrink breasts [126]. The juice of poison hemlock together with seeds of betony (*Stachys officinalis* (L.) Trevis. ex Briq.) and fennel (*Foeniculum vulgare* Mill.) mixed into wine were used for the treatment of the bite of a mad dog (rabies). The plant has been the last resort antidote for strychnine and other virulent poisons. Religious sects in the 1400s and 1500s used roasted roots to relieve pains of gout. From the 1760s onwards the plant was used as a cure for cancerous ulcers. Tinctures and extracts made from hemlock have been used as a sedative and an anodyne (analgesic). Its antispasmodic effects were used to treat tetanus, asthma, epilepsy, whooping cough, angina, chore and stomach pains [9,45,47,122,123,127]. In Finnish folk medicine, poison hemlock has been used as a powder, plaster and poultice to treat hardened glands, cramp and malignant wounds [128].

Dried leaf and juice of poison hemlock were part of the official London and Edinburgh pharmacopeias of 1864–1898. The last official recognition of the medical use of poison hemlock was in 1938 in the British Pharmaceutical Codex [111]. The reason for discontinuation appears to be the difficulty of manufacturing a medicine with even quality: different preparations varied in potency [86]. If poison hemlock is used internally it must be carefully administrated, as narcotic poisoning with paralysis and loss of speech may result from overdosage [9,47].

The coniine alkaloids could serve as a starting point for the synthesis of specific and less toxic spinal relaxants [111,121]. Bowman and Sangvi [111] noted that the hemlock alkaloids do not have a local anesthetic effect, or it is very weak. However, later research has found that coniine has a local anesthetic effect in mice and rats [122,123].

### 5.3. Toxicity to Animals

Poisoning of various mammals has been reported after feeding on poison hemlock: cattle (*Bos taurus* L.) [129,130,131,132], pigs (*Sus scrofa domesticus* Erxleben) [41,133,134,135,136,137,138,139], horses (*Equus ferus caballus* L.) [140,141,142], deer [132], tule elks (*Cervus canadensis nannodes*) [143], goats (*Capra aegagrus hircus* L.) [17,144], sheep (*Ovis aries* L.) [41,145] and rabbits (*Oryctolagus cuniculus* L.) [146]. Coniine is deadly to several birds: quails (*Coturnix corturnix* L.), chickens (*Gallus gallus* L.) and turkeys (*Meleagris gallopavo* L.) [147]. General signs of poison hemlock toxicosis on mammals are muscular weakness, incoordination, trembling, nervousness, ataxic gait, knuckling at the fetlock joints, excessive salivation, bloating, intestinal irritation, rapid and weak pulse, loss of appetite, cyanotic membranes, dilating pupils, initial central nervous system stimulation, then depression and finally death from respiratory paralysis (all symptoms not always present) [54,141,142]. Some of these signs appear within 1 h of consumption, followed by respiratory paralysis in 2–3 h, and some of the symptoms come later (in 3–4 days) [141,142]. The treatment for animals includes nerve and heart stimulants with large doses of mineral oil and purgatives to empty the digestive tract [142].

Different species have different reactions to coniine. Its toxicity varies from the lowest to the highest tolerance as follows: cows < mares < ewes [141]. For example, to cows, coniine is toxic in a daily dosage of 3.3–6.6 mg/kg [141] and about 1 kg of poison hemlock is deadly [142]. The lethal level for pigs is 1 g/kg of seeds and 8 g/kg of the plant [136]. Coniine, piperidine, and 2-ethylpiperidine are toxic to cattle, with classic symptoms of hemlock poisoning. 2-Methylpiperidine, 2-piperidine-ethanol, conyrine, 3-methylpiperidine and *N*-methylpiperidine are not toxic. Inhalation of coniine or crushed green plant material does not cause toxication [148]. The primary toxicants to livestock are coniine and γ-coniceine [54].

Animal toxications happen when there is no other vegetation available [57,149]. It has been observed that animals feeding on poison hemlock return to feed on other plants [8,144]. Even though, it is possible that hemlock alkaloids can be addictive to surviving animals.

### 5.4. Teratogenicity to Animals

Coniine and hence poison hemlock are teratogenic to animals (from the most intense to the least): cows > sows > ewes [145]. Piperidine, 2-methylpiperidine, 2-ethylpiperidine, 2-piperidineethanol, conyrine, 3-methylpiperidine and *N*-methylpiperidine are not teratogenic [148]. For a piperidine alkaloid to become teratogenic it must have at least three carbons in the ‘tail’ and only one or no double bonds in the ring structure [148]. The reason behind the teratogenic malformations is alkaloid-induced fetal immobilization [150]. The malformations sometimes resolve spontaneously after birth in 8–10 weeks. Poison hemlock plant material causes multiple congenital contractures and cleft palate. Multiple congenital contractures include torticollis, scoliosis, lordosis, arthrogryposis, rib cage anomalies, overextension, flexure and rigidity of joints [151].

### 5.5. Toxicity to Humans

A historic poem of *koneion* (hemlock) intoxication by Nicander of Colophon (204–135 B.C.) in Alexipharmaca [126] describes how the effects progress in the human body:

“Take note of the noxious draught which is hemlock, for this drink assuredly loses disaster upon the head bringing the darkness of night: the eyes roll, and men roam the streets with tottering steps and crawling upon their hands; a terrible choking blocks the lower throat and the narrow passage of the windpipe; the extremities grow cold; and in the limbs the stout arteries are contracted; for a short while the victim draws breath like one swooning, and his spirit beholds Hades”.

Reese’s [152] description is as follows: a headache, “imperfect vision”, pharyngeal pain, vomiting, drowsiness, gradual paralysis of extremities and death at last “from apnea”. Other symptoms may be convulsions, coma, violent delirium, salivation and involuntary discharges from the bladder and bowels. Modern major clinical effects on humans are irritation of oral mucosa, salivation, nausea, emesis, slight abdominal pain, diarrhea (uncommon), bradycardia, miosis, hypertension to tachycardia, hypotension, mydriasis, seizures following ascendant muscle paralysis and respiratory failure. Diagnosis uses blood gases, electrolytes and a plant sample. First-aid and management procedures are immediate gastric lavage or emesis following activated charcoal with a purgative drug. Treatment is mainly to ensure adequate respiratory function [45]. The important factor is to get the patient quickly into hospital care, as the poisoning symptoms appear quite rapidly. With the help of artificial breathing such as intubation, it is possible to save the patient’s life.

Coniine is toxic to humans and 3 mg produce symptoms. Up to 150–300 mg coniine can be tolerated, which translates to 6–8 leaves (6 g) [45,153,154]. Accidental ingestion of poisonous plants (e.g., poison hemlock) can also be sourced to herbal medication that has been incorrectly prepared or an incorrect plant has been used due to misidentification [155].

### 5.6. Socrates

Socrates died in 399 B.C. He was sentenced to death because of corrupting the youth of Athens and failing to recognize the city’s traditional gods. The sentencing was passed with only a small majority. The execution was carried out with a dose of poison potion called *pharmakon* according to the tradition. The death of Socrates is described in Plato’s Socratic dialogue, Phaedo [127,156].

Often the poison that killed Socrates has been suggested to have been poison hemlock [157]. Plato’s description of the death of Socrates might be true based on the results of research by Arihan et al. [122], according to which coniine has antinociceptive activity. It may have been a mixture of *koneion* (poison hemlock) and for example opium, myrrh, and wine [121,122,126,127,156,157]. Correctly performed the poison, as in Plato’s description, would speed the death [127]. Theophrastus’ Enquiry into Plants describes how to prepare hemlock for quick and painless death; poppy and other similar herbs are mixed with it. However, there is no information concerning which hemlock plant the recipe uses, poison hemlock (*C. maculatum*), water hemlock (*Cicuta* sp.) or water dropwort (*Oenanthe crocata* L.) [158].

There are several factors supporting the theory that poison hemlock was one of the components of the poison administered to Socrates. The paralysis started from the feet, death was due to respiratory failure, the feeling of “cold” and “stiffness” was in the calves and spread upwards to the chest, the loss of feeling in the legs and the fact that death appears to have been quite quick on the basis of the dialogue. The contradictions for poison hemlock are that there was no abdominal pain, nausea, vomiting or diarrhea [157]. A Scottish physician, John Harley, tested poison hemlock on himself in the 19th century and his description agrees rather well with Plato’s [126]. When coniine and opium are used together and tested on rats, the effects of the mixture are quicker than either alone. The symptoms are depression of respiration, very strong cyanosis, paralyzed skin sensitivity, almost absent convulsions, strong muscle paralysis, pronounced paralyzing action of coniine and lowered anesthetic power of opium. Opium and poison hemlock were used together to speed up the death of Socrates according to de Boer [121]. It could explain the skin effects in the legs and the last-minute speaking of Socrates. Poison hemlock alone would have needed administration of quite a large amount of the plant to reach the necessary dose [121].

Nevertheless, it is possible that the description of Socrates’ death has some artistic license and is not entirely accurate [127], or there might be some confusion in the description of symptoms, as Plato’s writing is in fact a quotation of Crito [156,157]. It is possible that Plato wanted to present a beautified picture of Socrates’ passing without all the gruesome details [156]. Bloch [126], however, concluded that Plato described the poisoning of Socrates correctly once all the confusing layers are peeled away. Sullivan [158] supported the notion that poison hemlock was behind the poisoning of Socrates without a poetic license, as the description in Phaedo is quite clear.

## 6. Ecological Role of Hemlock Alkaloids

Coniine is one of the floral scent compounds in poison hemlock [51]. Nitao [159] noted that flies (Diptera) pollinate poison hemlock flowers in large numbers, and thus could be attracted by coniine. Volatile alkaloids are part of the host plant recognition of the hemlock moth [160]. The larvae of the hemlock moth also need coniine in order to develop properly [160]. As attacked plants produce more alkaloids, moth larvae must therefore have a peculiar strategy to handle the alkaloids [8,38].

An important question is the function of coniine in *Sarracenia* sp., since the plants live in nutrient-poor environments, and nitrogen-consuming compounds would be costly without benefits. However, Butler and Ellison [161] studied nitrogen acquisition of *S. purpurea* and observed that actually the plant’s pitchers are quite efficient in prey capture, and thus could greatly enhance the available nitrogen for the following growth season. Mody et al. [6] postulated that coniine could be an insect-paralyzing agent because *S. flava*, in which they found coniine, paralyzed fire ants, and Harborne [73] suggested that coniine attracts insects into the pitcher. When insect attracting and paralyzing are taken into account, investment in coniine biosynthesis by a *Sarracenia* plant could indicate that coniine is an enhancing mechanism for prey capture due to its dual role.

## 7. Conclusions

All in all, there has been tremendous progress in the research on hemlock alkaloids; especially recent advances in the biosynthetic pathway elucidation have helped to clarify the carbon backbone formation. Now, almost all pathway enzymes are identified except for one critical reduction step. This putative polyketide reductase would remove the C-5 keto group from the polyketide intermediate. It is expected that the gene encoding this enzyme as well as the genes for the known enzymes in the pathway will be soon revealed by sequencing approaches. The renewed interest in the pharmaceutical utilization of hemlock alkaloid derivatives would make these efforts worthwhile.

## Figures and Tables

**Table 1:** Poison hemlock names in selected countries.

Language	Name
Argentina	cicuta, denta
Belgium	dolle kervel, gevlekte scheerling
Brazil	cicuta, cicuta da europa, cigue, cuquta maior, funcho selvagem
Chile	cicuta, sarrac
Denmark	skarntyde
Finland	myrkkykatko
France	grande cique
Germany	Gefleckter Schierling
Italy	cicuta maggiore
Japan	doku-ninjin
Portugal	ansarina-malhada
Spain	perejillon cicuta
Sweden	odört
Turkey	tri baldiran

**Table 2:** Hemlock alkaloids reported from *Aloe* [1,2,3,4].

*Aloe* Species	Alkaloids
*A. ballyii* Reynolds	γ-coniceine, conhydrinone
*A. deltoideodonta* Baker	γ-coniceine, a trace of pseudoconhydrine
*A. descoingsii* Reynolds	coniine, conhydrine
*A. gariepensis* Pillans	γ-coniceine, conhydrine, a trace of coniine and *N*-methylconiine
*A. globuligemma* Pole Evans	γ-coniceine, coniine, conhydrine, *N*-methylconiine
*A. gracilicaulis* Reynolds and P.R.O. Bally	γ-coniceine
*A. ibitiensis* Perrier	γ-coniceine
*A. krapholiana* Marloth.	coniine, conhydrine
*A. ortholopha* Christian and Milne-Redh.	coniine, conhydrine
*A. ruspoliana* Baker	γ-coniceine
*A. sabaea* Schweinf. (syn. *A. gillilandii* Reynolds)	γ-coniceine, coniine, *N*,*N*-dimethylconiine
*A. viguieri* Perrier	coniine, γ-coniceine, *N*-methylconiine

**Table 3:** The half maximal effective concentration (EC_50_) of cell lines expressing nAChR in vitro for different alkaloids and their enantiomers [110,119].

	EC_50_ of the Cell Line Expressing nAChR	
Alkaloid	TE-671	SH-SY5Y
(−)-coniine	115 μM	9.6 μM
(±)-coniine	208 μM	51.4 μM
(+)-coniine	900 μM	10.2 μM
γ-coniceine	1.3 μM	-
(−)-*N*-methylconiine	105 μM	-
(±)-*N*-methylconiine	405 μM	-
(+)-*N*-methylconiine	3000 μM	-
